# Three-year emergency medicine training program in The Netherlands: first evaluation from the residents’ perspective

**DOI:** 10.1186/1865-1380-6-30

**Published:** 2013-07-26

**Authors:** Salomon Willem Koning, Menno Iskander Gaakeer, Rebekka Veugelers

**Affiliations:** 1 Emergency Medicine, Emergency Medicine Department, University Medical Center Utrecht, Heidelberglaan 100, Utrecht, CX 3584, The Netherlands

**Keywords:** Education [MESH], Emergency medicine [MESH], The Netherlands [MESH], Medical staff, Hospital [MESH], Evaluation studies as topic [MESH]

## Abstract

**Background:**

The Netherlands’ 3-year training in Emergency Medicine (EM) was formally approved and introduced in November 2008. To identify areas for improvement, we conducted the first evaluation of this curriculum from the residents’ perspective.

**Methods:**

A questionnaire was composed on ten aspects of the curriculum. It contained multiple-choice, open and opinion questions; answers to the latter were classified using the Likert scale. The questionnaires were mailed to all enrolled residents.

**Results:**

We mailed questionnaires to all 189 enrolled residents, and 105 responded (55.6%). Although they were satisfied with their training overall, 96.2% thought it was currently too short: 18.3% desired extension to 4 years, 76.0% to 5 and 1.9% to 6 years. Nevertheless, residents expected that they would function effectively as emergency physicians (EPs) after finishing their 3-year training program. Bedside teaching was assessed positively by 35.2%. All rotations were assessed positively, with the general practice rotation seen as contributing the least to the program. According to 43.7%, supervising EPs were available for consultation; 40.7% thought that, in a clinical capacity, the EP was sufficiently present during residents’ shifts. When EPs were present, 82.5% found them to be easily accessible, and 66.6% viewed them as role models. In the Emergency Medicine Departments (EDs) with a higher number of EPs employed, residents tended to perceive better supervision and were more likely to see their EPs as role models. While residents were stimulated to do research, actual support and assistance needed to be improved.

**Conclusion:**

Although overall, the current training program was evaluated positively, the residents identified four areas for improvement: (1) in training hospitals, trained EPs should be present more continuously for clinical supervision; (2) bedside teaching should be improved, (3) scientific research should be facilitated more and (4) the training program should be extended.

## Background

The Netherlands currently has a 3-year national emergency medicine training program. Its length is the result of a compromise: in 1999, The Netherlands Society of Emergency Physicians (NVSHA) attempted to introduce an innovative national emergency medicine (EM) program based on a 5-year curriculum. As this attracted considerable opposition from the existing specialties, the original plans for a comprehensive 5-year program were reduced to the current 3-year curriculum. This was eventually adopted in November 2008 [1,2] when the Medical Specialist Registration Committee (MSRC) of the Royal Dutch Medical Association (KNMG) recognized the EM program [3]. Today however, EM is still not recognized as a medical specialty [3].

The Netherlands currently has 86 hospital organizations. Between them, these have 96 emergency departments (EDs), 27 of which are accredited as training hospitals for emergency medicine [1]. Non-accredited EM-training hospitals have no EM training at all. The numbers of ED visits per year per hospital range between 10,000 and 50,000. In 2009 there were an estimated 2.2 million ED visits in The Netherlands [4].

In Dutch medical education, students become medical doctors after 6 years of medical training, 2 to 3 years of which consists of clinical rotations. Although junior doctors can then apply for the EM resident training, most work for 1 or 2 years in emergency medicine or another specialty without starting a training program. Roughly half of the current 3-year EM curriculum (approximately 18 months) consists of ED rotations. The rest is spent on rotations elsewhere. Six rotations are obligatory: cardiology, intensive care, pediatrics, anesthesiology, pre-hospital ambulance care and general practice. The length of rotations is not uniform between training hospitals. Other rotations are optional and differ between training hospitals. Residents participate in a yearly national progress examination, but there are no final board examinations.

To identify areas for improvement, this article describes the first evaluation of The Netherlands’ current 3-year EM training program to be conducted from a residents’ perspective.

## Methods

To conduct a survey among EM residents, we used a questionnaire addressing ten main aspects of the curriculum: respondents’ general data, hospital setting, program length, overall workload, training content, rotations, self-image, scientific research, examinations, supervision by an experienced emergency physician (EP) and the extent to which EPs were regarded as role models. We based these questions not only on the Dutch Curriculum of EM [5], but also on the CanMED roles [6], an educational framework that identifies and describes seven roles defining an optimally prepared physician: those of medical expert, communicator, collaborator, manager, health advocate, scholar and professional.

Opinion question could be answered on a five-point Likert scale. Other questions had open or multiple-choice answers.

The questionnaire was mailed on 20 December 2011. It was anonymous but for a unique personal number that was accessible only to the main investigator and used exclusively for mailing purposes. A return envelope was included. Non-responders were sent a reminder on 16 January 2012.

We included currently enrolled emergency residents. All returned questionnaires were processed manually in an SPSS database by the main investigator. SPSS 15 was used for the statistical calculations. To investigate differences between research in a university hospitals and non-university hospitals, Student’s *t*-test was performed.

## Results

### General data

There were 189 enrolled EM residents, 73.0% of whom (134) were female and 74.1% of whom (140) were enrolled in non-university training hospitals. Their distribution per year was essentially equal.

We mailed questionnaires to all EM residents, and 105 responded, 81 before the reminder and 24 after it. Table 1 shows the respondents’ general data and their type of training hospital.

**Table 1:** Respondents’ general data

		***n***	**%**
Number of respondents		105	55.6
Gender	Female	78	74
	Male	27	26
University hospital		25	24.8
Responses per training year	1st year	23	21.9
	2nd year	31	29.5
	3rd year	51	48.5
Years of experience at the ED prior to the training program	None	10	9.5
	1 year	34	32.3
	2 years	38	36.1
	> 2 years	22	20.9
Years of experience as a physician elsewhere prior to the training program	None	34	32.3
	1 year	28	26.7
	Two years	24	22.9
	> 2 years	18	17.1

In total, 66.3% agreed that the development of EM was a priority in their hospital.

### Reflection on training length

While 3.8% residents were content with the current 3-year structure, 96.2% believed it should be extended, with 18.3% desiring extension to 4 years, 76.0% to 5 years and 1.9% to 6 years.

### Workload, education and rotations

A Dutch full-time residency contract comprises 48 h/week, 10 h/week of which should be spent on learning (including courses, teaching days and learning on the job). Most residents (82.8%) were positive about the number of working hours, and 82.9% gave a positive appraisal to the variety of diseases and conditions presented and the number of patients seen during an ED shift. Residents reported spending an average of 3 h 40 min a week on self-access study, which 42.9% regarded as enough. In total, 74% were positive about their increases in knowledge during the training. Although they were also positive about most aspects of the curriculum (i.e., the local, regional and national training days), only 35.2% were positive about the received bedside teaching (Table 2). Although they evaluated their six mandatory rotations positively, they were neutral toward their general practitioner rotation (Table 2).

**Table 2:** Education and rotations

	**Very poor (%)**	**Poor (%)**	**Neutral (%)**	**Good (%)**	**Very good (%)**	Completed**
Bedside teaching (%)	9 (8.6)	27 (25.7)	**33 (31.4)**	30 (28.6)	6 (5.7)	NA
Local education (%)	0 (0)	80 (7.7)	13 (12.5)	**56 (53.8)**	27 (26.0)	NA
Regional education (%)	0 (0)	6 (5.8)	20 (19.2)	**70 (67.3)**	8 (7.7)	NA
National education (%)	0 (0)	5 (4.8)	31 (29.8)	**65 (62.5)**	3 (2.9)	NA
Emergency department rotation	0 (0)	0 (0)	2 (1.9)	24 (23.3)	**77 (74.8)**	103
Anesthesiology rotation	0 (0)	1 (1.1)	13 (13.8)	**49 (52.1)**	31 (33.0)	94
Intensive care rotation	0 (0)	0 (0)	8 (9.2)	33 (37.9)	**46 (52.9)**	87
General practice rotation	1 (2.4)	8 (19.0)	**15 (35.7)**	14 (33.3)	4 (9.5)	42
Pediatric rotation	0 (0)	2 (3.3)	6 (9.8)	**31 (50.8)**	22 (36.1)	61
EMT^*^ rotation	1 (1.8)	0 (0)	13 (23.2)	**25 (44.6)**	17 (30.4)	56
Cardiology rotation	0 (0)	0 (0)	8 (9.0)	**43 (48.3)**	38 (42.7)	89

*Emergency medical teams or ambulance; **residents who completed the rotation; NA not applicable. Numbers in bold represent the majority of the group.

### Mastering endpoints as an emergency physician

The training program specifies 18 themes that residents should master. Table 3 shows how the residents viewed their competency with regard not only to these themes, but also to six specific ED competencies distributed over each training year. As future EPs, residents were positive with regard to mastering 14 out of the 18 themes and 4 of the 6 ED competences. In four themes (dermatology, psychiatry, pre-hospital care and knowledge/research), they had a more neutral view of their competency. With regard to two specific ED competences, 19.0% believed themselves to be capable of performing a focused assessment with sonography in trauma (FAST), and 45.7% believed themselves to be capable of performing procedural sedation (PSA) and analgesia. The values did not differ substantially per year, and as no relevant differences could have been expected with such a small group, we did no statistical significance tests.

**Table 3:** Residents’ image of their future performance as emergency physicians

		**Very poor (%)**		**Poor (%)**		**Neutral (%)**		**Good (%)**		**Very good (%)**	
		**Year**	**Overall**	**Year**	**Overall**	**Year**	**Overall**	**Year**	**Overall**	**Year**	**Overall**
**Anticipated mastery of themes**											
Airway	1st	0 (0)	0 (0)	1 (4.3)	3 (2.9)	7 (30.4)	25 (23.8)	**12 (52.2)**	**66 (62.9)**	3 (13.0)	11 (10.5)
	2nd	0 (0)		1 (3.2)		8 (25.8)		**18 (58.1)**		4 (12.9)	
	3rd	0 (0)		1 (2.0)		10 (19.6)		**36 (70.6)**		4 (7.8)	
Breathing	1st	0 (0)	0 (0)	0 (0)	1 (1.0)	3 (13.0)	6 (5.7)	**14 (60.9)**	**80 (76.2)**	6 (26.1)	18 (17.1)
	2nd	0 (0)		1 (3.2)		1 (3.2)		**24 (77.4)**		5 (16.1)	
	3rd	0 (0)		0 (0)		2 (3.9)		**42 (82.4)**		7 (13.7)	
Circulation	1st	0 (0)	0 (0)	0 (0)	0 (0)	1 (4.3)	6 (5.7)	**16 (69.6)**	**77 (73.3)**	6 (26.1)	22 (21.0)
	2nd	0 (0)		0 (0)		1 (3.2)		**22 (71.0)**		8 (25.8)	
	3rd	0 (0)		0 (0)		4 (7.8)		**39 (76.5)**		8 (15.7)	
Disability	1st	0 (0)	1 (1.0)	0 (0)	2 (1.9)	3 (13.0)	17 (16.2)	**20 (87.0)**	**77 (73.3)**	0 (0)	8 (7.6)
	2nd	1 (3.2)		0 (0)		4 (12.9)		**24 (77.4)**		2 (6.5)	
	3rd	0 (0)		2 (3.9)		10 (19.6)		**33 (64.7)**		6 (11.8)	
Exposure/environment	1st	0 (0)	0 (0)	0 (0)	4 (3.8)	3 (13.0)	15 (14.3)	**19 (82.6)**	**75 (71.4)**	1 (4.3)	11 (10.5)
	2nd	0 (0)		1 (3.2)		5 (16.1)		**24 (77.4)**		1 (3.2)	
	3rd	0 (0)		3 (5.9)		7 (13.7)		**32 (62.7)**		9 (17.6)	
Secondary assessment	1st	0 (0)	0 (0)	0 (0)	1 (1.0)	1 (4.3)	5 (4.8)	**16 (69.6)**	**81 (77.1)**	6 (26.1)	18 (17.1)
	2nd	0 (0)		1 (3.2)		3 (9.7)		**22 (71.0)**		5 (16.1)	
	3rd	0 (0)		0 (0)		1 (2.0)		**43 (84.3)**		7 (13.7)	
Facial injuries	1st	0 (0)	0 (0)	2 (8.7)	9 (8.6)	9 (39.1)	33 (31.4)	**10 (43.5)**	**59 (56.2)**	2 (8.7)	4 (3.8)
	2nd	0 (0)		2 (6.5)		9 (29.0)		**19 (61.3)**		1 (3.2)	
	3rd	0 (0)		5 (9.8)		15 (29.4)		**30 (58.8)**		1 (2.0)	
Internal medicine	1st	0 (0)	0 (0)	1 (4.3)	3 (2.9)	3 (13.0)	20 (19.0)	**16 (69.6)**	**72 (68.6)**	3 (13.0)	10 (9.5)
	2nd	0 (0)		0 (0)		6 (19.4)		**21 (67.7)**		4 (12.9)	
	3rd	0 (0)		2 (3.9)		11 (21.6)		**35 (68.6)**		3 (5.9)	
Dermatology	1st	0 (0)	2 (1.9)	8 (34.8)	36 (34.3)	**12 (52.2)**	**44 (41.9)**	3 (13.0)	23 (21.9)	0 (0)	0 (0)
	2nd	2 (6.5)		9 (29.0)		**13 (41.9)**		7 (22.6)		0 (0)	
	3rd	0 (0)		19 (37.3)		**19 (37.3)**		13 (25.5)		0 (0)	
Muscular/skeletal	1st	0 (0)	0 (0)	0 (0)	0 (0)	2 (8.7)	(7.6)8	**14 (60.9)**	**64 (61.0)**	7 (30.4)	33 (31.4)
	2nd	0 (0)		0 (0)		3 (9.7)		**21 (67.7)**		7 (22.6)	
	3rd	0 (0)		0 (0)		3 (5.9)		**29 (56.9)**		19 (37.3)	
Psychiatry	1st	0 (0)	0 (0)	2 (8.7)	13 (12.4)	**14 (60.9)**	**62 (59.0)**	7 (30.4)	30 (28.6)	0 (0)	0 (0)
	2nd	0 (0)		3 (9.7)		**20 (64.5)**		8 (25.8)		0 (0)	
	3rd	0 (0)		8 (15.7)		**28 (54.9)**		15 (29.4)		0 (0)	
General practitioner	1st	0 (0)	0 (0)	0 (0)	1 (1.0)	6 (26.1)	31 (29.8)	**15 (65.2)**	**62 (59.6)**	2 (8.7)	10 (9.6)
	2nd	0 (0)		0 (0)		14 (46.7)		**15 (50.0)**		1 (3.3)	
	3rd	0 (0)		1 (2.0)		11 (21.6)		**32 (62.7)**		7 (13.7)	
Geriatrics	1st	0 (0)	0 (0)	4 (17.4)	8 (7.6)	9 (39.1)	44 (41.9)	**10 (43.5)**	**51 (48.6)**	0 (0)	2 (1.9)
	2nd	0 (0)		0 (0)		**17 (54.8)**		14 (45.2)		0 (0)	
	3rd	0 (0)		4 (7.8)		18 (35.3)		**27 (52.9)**		2 (3.9)	
Pediatrics	1st	0 (0)	0 (0)	1 (4.3)	9 (8.6)	5 (21.7)	21 (20.0)	**16 (69.6)**	**72 (68.6)**	1 (4.3)	3 (2.9)
	2nd	0 (0)		4 (12.9)		6 (19.4)		**21 (67.7)**		0 (0)	
	3rd	0 (0)		4 (7.8)		10 (19.6)		**35 (68.6)**		2 (3.9)	
Pain/sedation	1st	1 (4.3)	2 (1.9)	3 (13.0)	13 (12.4)	2 (8.7)	21 (20.0)	**12 (52.2)**	**52 (49.5)**	5 (21.7)	17 (16.2)
	2nd	0 (0)		5 (16.1)		9 (29.0)		**15 (48.4)**		2 (6.5)	
	3rd	1 (2.0)		5 (9.8)		10 (19.6)		**25 (49.0)**		10 (19.6)	
Pre-hospital care	1st	1 (4.3)	1 (1.0)	3 (13.0)	25 (23.8)	**12 (52.2)**	**40 (38.1)**	6 (26.1)	35 (33.3)	1 (4.3)	4 (3.8)
	2nd	0 (0)		12 (38.7)		**13 (41.9)**		6 (19.4)		0 (0)	
	3rd	0 (0)		10 (19.6)		15 (29.4)		**23 (45.1)**		3 (5.9)	
Traumatology	1st	0 (0)	0 (0)	0 (0)	0 (0)	2 (8.7)	7 (6.7)	**14 (60.9)**	**65 (61.9)**	7 (30.4)	33 (31.4)
	2nd	0 (0)		0 (0)		3 (9.7)		**20 (64.5)**		8 (25.8)	
	3rd	0 (0)		0 (0)		2 (3.9)		**31 (60.8)**		18 (35.3)	
Knowledge/research	1st	0 (0)	0 (0)	1 (4.3)	9 (8.6)	**16 (69.6)**	**51 (48.6)**	5 (21.7)	41 (39.0)	1 (4.3)	4 (3.8)
	2nd	0 (0)		3 (9.7)		**14 (45.2)**		12 (38.7)		2 (6.5)	
	3rd	0 (0)		5 (9.8)		21 (41.2)		**24 (47.1)**		1 (2.0)	
**Anticipated mastery of ED competences**											
I can function independently as a EP	1st	0 (0)	0 (0)	0 (0)	2 (1.9)	3 (13.0)	13 (12.4)	**16 (69.6)**	**73 (69.5)**	4 (17.4)	17 (16.2)
	2nd	0 (0)		1 (3.2)		4 (12.9)		**23 (74.2)**		3 (9.7)	
	3rd	0 (0)		1 (2.0)		6 (11.8)		**34 (66.7)**		10 (19.6)	
I can care for an unstable patient	1st	0 (0)	0 (0)	0 (0)	0 (0)	1 (4.3)	8 (7.6)	**16 (69.6)**	**70 (66.7)**	6 (26.1)	27 (25.7)
	2nd	0 (0)		0 (0)		2 (6.5)		**23 (74.2)**		6 (19.4)	
	3rd	0 (0)		0 (0)		5 (9.8)		**31 (60.8)**		15 (29.4)	
I can care for a trauma patient	1st	0 (0)	0 (0)	1 (4.3)	2 (1.9)	1 (4.3)	7 (6.7)	**11 (47.8)**	**65 (61.9)**	10 (43.5)	31 (29.5)
	2nd	0 (0)		1 (3.2)		4 (12.9)		**21 (67.7)**		6 (19.4)	
	3rd	0 (0)		1 (2.0)		2 (3.9)		**33 (64.7)**		15 (29.4)	
I can lead a cardiopulmonary resuscitation	1st	1 (4.3)	3 (2.9)	3 (13.0)	7 (6.7)	2 (8.7)	7 (6.7)	**7 (30.4)**	**54 (51.4)**	10 (43.5)	34 (32.4)
	2nd	1 (3.2)		1 (3.2)		3 (9.7)		**20 (64.5)**		6 (19.4)	
	3rd	1 (2.0)		3 (5.9)		2 (3.9)		**27 (52.9)**		18 (35.3)	
I can perform a focused assessment of sonography in trauma	1st	4 (17.4)	27 (25.7)	**11 (47.8)**	**42 (40.0)**	4 (17.4)	16 (15.2)	3 (13.0)	16 (15.2)	1 (4.3)	4 (3.8)
	2nd	6 (19.4)		**9 (29.0)**		7 (22.6)		8 (25.8)		1 (3.2)	
	3rd	17 (33.3)		**22 (43.1)**		5 (9.8)		5 (9.8)		2 (3.9)	
I can perform procedural sedation and analgesia	1st	2 (8.7)	10 (9.5)	4 (17.4)	28 (26.7)	5 (21.7)	19 (18.1)	**9 (39.1)**	**33 (31.4)**	3 (13.0)	15 (14.3)
	2nd	4 (12.9)		9 (29.0)		6 (19.4)		**11 (35.5)**		1 (3.2)	
	3rd	4 (7.8)		**15 (29.4)**		8 (15.7)		13 (25.5)		11 (21.6)	

Numbers in bold represent the majority of the group.

### Research

In The Netherlands, scientific research is one of the themes specified in the medical training curriculum. While 73.3% of the residents agreed that they were stimulated to perform research in their training hospital, only 52.9% felt that they were actually supported in doing so. Residents at university hospitals felt significantly more supported in their research (*p* < 0.05). Table 4 shows how meaningful they found scientific research and also indicates the kind of research they were already doing.

**Table 4:** Research

		**Extent of agreement with the statement “research is meaningful”**					
		**Completely disagree (%)**	**Disagree (%)**	**Neutral (%)**	**Agree (%)**	**Completely agree (%)**	**Performed (%)**
Local research	General research	6 (5.7)	13 (12.4)	25 (23.8)	**54 (51.4)**	7 (6.7)	57 (54.3)
	CAT*	2 (1.9)	11 (10.5)	15 (14.3)	**68 (64.8)**	9 (8.6)	96 (91.4)
	Oral presentations	0 (0)	3 (2.9)	11 (10.6)	**78 (75.0)**	12 (11.5)	95 (90.5)
National	Poster presentation	1 (1.0)	11 (10.6)	24 (23.1)	**64 (61.5)**	4 (3.8)	53 (50.5)
	Oral presentation	1 (1.0)	11 (10.5)	24 (22.9)	**64 (61.0)**	5 (4.8)	23 (21.9)
	Publication	2 (1.9)	22 (21.0)	34 (32.4)	**43 (41.0)**	4 (3.8)	12 (11.4)
International	Poster presentation	3 (2.9)	21 (20.0)	**38 (36.2)**	**38 (36.2)**	5 (4.8)	9 (8.6)
	Oral presentation	2 (1.9)	20 (19.0)	33 (31.4)	**40 (38.1)**	10 (9.5)	1 (1.0)
	Publication	3 (2.9)	26 (24.8)	**36 (34.3)**	32 (30.5)	8 (7.6)	13 (12.4)

*Critical appraisal of a topic. Numbers in bold represent the majority of the group.

While overall residents agreed that doing research is meaningful, publication in a Dutch journal had been achieved by 11.4% of the residents and publication in an international journal by 12.4%.

### Examination

During the training, EPs use various forms to assess residents’ day-to-day performance; these assesse technical skills, oral presentations, patient handovers and bedside observation of a physical examination, for example. There is a yearly national progress test and no official board exam. Overall, 66.7% agreed that this represented a meaningful and representative way of testing the objectives of the national EM training program. Residents keep a portfolio – an activity that 60.7% valued as meaningful. As well as assessment forms, the portfolio includes the results of the national progress test and of a personal training plan. How residents viewed the different methods of their assessment is shown in Table 5.

**Table 5:** The following examination methods are meaningful

	**Completely disagree (%)**	**Disagree (%)**	**Neutral (%)**	**Agree (%)**	**Completely agree (%)**
Short clinical assessment^*^	1 (1.0)	4 (3.8)	23 (21.9)	**65 (61.9)**	12 (11.4)
360 Degree assessment^**^	3 (2.9)	13 (12.4)	20 (19.0)	**58 (55.2)**	11 (10.5)
OSATS^***^	0 (0)	4 (3.8)	13 (12.4)	**79 (75.2)**	9 (8.6)
CATs^****^	1 (1.0)	15 (14.3)	26 (24.8)	**59 (56.2)**	4 (3.8)
Oral presentations	0 (0)	7 (6.7)	23 (21.9)	**72 (68.6)**	3 (2.9)
Progress test	3 (2.9)	13 (12.4)	29 (27.6)	**52 (49.5)**	8 (7.6)
Personal training plan	1 (1.0)	0 (0)	**48 (46.2)**	37 (35.6)	4 (3.8)
Self-reflection report	9 (8.6)	25 (23.8)	32 (30.5)	**35 (33.3)**	4 (3.8)
Progress and assessment interview^*****^	0 (0)	3 (2.9)	12 (11.4)	**64 (61)**	26 (24.8)

*Assessment of soft skills and knowledge; **combined feedback from patient, nurse, other residents, EP and other specialist; ***on-site assessment and training or assessment of technical skills; ****critical appraisal of a topic; *****interview with program director. Numbers in bold represent the majority of the group.

### Supervision, consultation and role model

Before the emergency medicine training program was developed, residents from other specialties working in the ED were supervised by a medical specialist in their specialty. Residents called a medical specialist to the ED when supervision was needed. The medical specialist were however usually not immediately available. Since the introduction of EPs to EDs, this problem has been solved in hospitals that have 24/7 EP coverage. However, most Dutch EM training hospitals do not have enough EPs to provide such coverage. In the meantime, whenever no EP is present, EM residents are supervised by medical specialist from other specialties. Table 6 shows who residents turn to for supervision and consultation.

**Table 6:** Supervision and consultation

		**No (%)**	**Yes (%)**
Consultations when emergency physician was not available	Consultation with emergency medicine resident	56 (56.0)	44 (44.0)
	Consultation with other resident other specialty	30 (29.4)	72 (70.6)
	Consultation with a medical specialist other specialty	19 (18.6)	83 (81.4)
Consultations when emergency physician was available	Consultation with emergency medicine resident	88 (86.3)	14 (13.7)
	Consultation with other resident other specialty	72 (70.6)	30 (29.4)
	Consultation with a medical specialist other specialty	37 (36.3)	65 (63.7)
	Consultation with emergency physician	5 (4.9)	97 (95.1)

**Figure 1:**
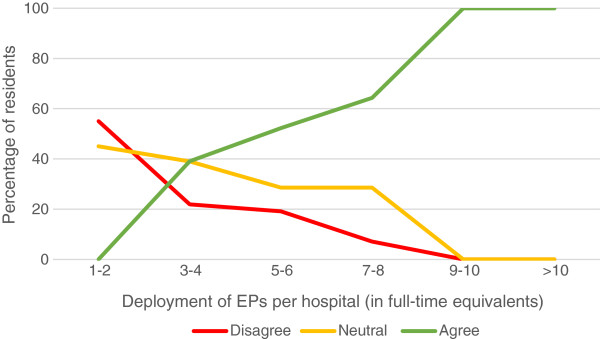
Resident’ agreement with the statement “I was satisfied with the presence of my EP in a clinical capacity during my shifts”.

**Figure 2:**
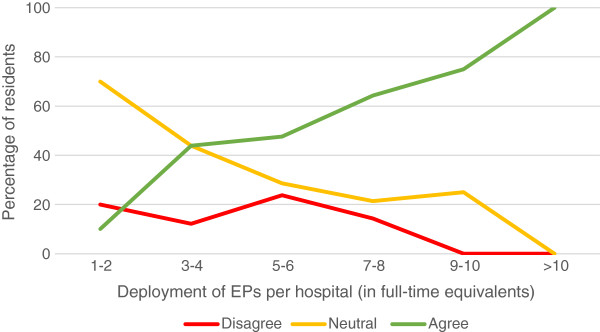
Resident’ agreement with the statement “The emergency physician had enough time to supervise me”.

**Figure 3:**
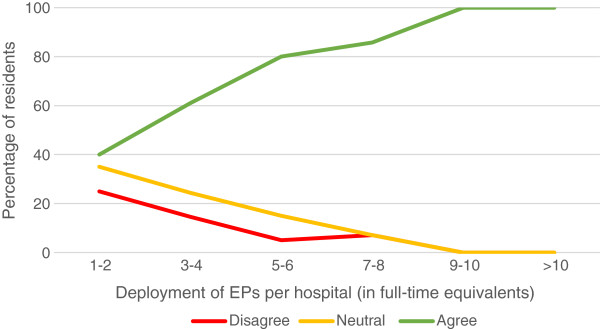
Resident’ agreement with the statement “I regard my emergency physician as a role model”.

In the view of 40.7% of respondents, an EP was present often enough during their shifts to provide supervision; 43.7% felt that EPs were available enough to provide consultation; 82.5% found the EP supervisor to be easy accessible. A third of the residents (66.6%) saw their EPs as a role model. If their hospital employed a higher number of EPs, residents were more satisfied with the presence and availability of EPs (Figure 1). There was no clear trend seen in satisfaction with bedside teaching and a higher number of employed EPs. However, they were more satisfied with the time EPs had to supervise their work (Figure 2) and were more likely to see an EP as a role model (Figure 3) if their hospital employed a higher number of EPs.

## Discussion

The Netherlands’ national curriculum for the EM training program was recognized in November 2008. Our survey found that although the residents believed that it provided them with a solid foundation, improvements were needed in several areas. One major improvement would be to conform to the 5-year curriculum originally proposed by the EuSEM [7]: in terms of both content and length, this would achieve a more uniform program. Similarly, if a higher number of EPs were employed in training hospitals, supervision and clinical presence could be improved.

This survey reflects the opinion of the residents in all training years and all training hospitals in The Netherlands. At 55.6%, the response rate was adequate.

Since no validated questionnaire was available to evaluate this training program, we developed our own on the basis of the CanMED roles and the Dutch curriculum. We used a Likert scale because it is a universal method that is both quantifiable and easily understood. It also allows participants to indicate a degree of agreement in a way that does not force them into a yes or no answer. We nonetheless acknowledge that, even if an extreme option would have been more accurate, this is a one-dimensional method that induces participants to concentrate on only one side of a response (i.e., agree or disagree) out of a desire to avoid the negatively associated extremes associated with extreme opinions. While great attention was paid to avoiding questions that were open to interpretation, we conclude that some questions could now be adjusted to further reduce the risk of misinterpretation.

Most EM residents (92%) felt that their 3-year training period should be extended. Seventy-eight percent believed it should be extended to at least 5 years. This is in agreement with the 5 years recommended by the EuSEM task force, which produced a guideline for the further development of EM curricula across Europe [7].

In countries where EM practice is established, training in EM takes place in the ED and is provided by qualified EPs. However, in the countries in which EM is still developing, more of the relevant knowledge and skills are obtained during rotations within other specialties. In the study by Aksay et al., Turkish EM residents described cardiology, anesthesiology and internal medicine as their most important rotations [8]. Our survey showed that the general practice rotation was felt to contribute the least to the training program. If the training period is extended to 5 years, the number, type and length of rotations should be evaluated.

Even though research and publications are essential to positioning EM as a fully qualified specialty, residents lag behind in a research role. While they were stimulated to undertake research, they received very little support. This might also lead to higher research productivity, as described by Ahmad et al. [9].

According to the EuSEM guideline [7], residents should be assessed on the basis of a portfolio that documents their theoretical, clinical and practical experiences. This should be checked yearly and should also include the residents’ written, oral and practical examinations. After completion, the portfolio should be submitted to the program director. A final individual assessment should include a final formal examination (written, oral and practical). In the Dutch situation, all these methods are integrated, but there is no final board examination. To adhere to the European standards, the introduction of a final board examination should be considered.

Bedside teaching and supervision are both known to be difficult issues in the ED, especially in overcrowded EDs [10]. Many studies have shown that residents’ learning and patient outcome both benefit from well-structured and well-organized bedside teaching and supervision [10-15]. Only 35% of the residents assessed the bedside teaching as good, but there was no clear trend seen with a higher number of EPs employed. Residents tended to be more positive about EPs’ presence in the clinic and the length of time they were supervised by them (Figure 1 and 2) when a higher number of EPs was employed. This suggests that training hospitals should provide 24/7 coverage by EPs.

A keystone of the Dutch [5] and European curriculum is Procedural Sedation and Analgesia [7]. Various articles have described the many facets of PSA performed by EPs [16-20], including a Dutch study showing that Dutch EPs can perform PSA safely [21]. The fact that most residents in our survey did not feel comfortable performing PSA nonetheless suggests that PSA should be given a more prominent role in the Dutch curriculum.

Although several training hospitals have started implementing Focused Assessment with Sonography in Trauma, this skill has not yet been established in the current Dutch curriculum. Unsurprisingly, the vast majority of residents currently enrolled did not feel comfortable performing it. There is sufficient international evidence to suggest that an ultrasound course for emergency medicine residents can be implemented successfully [22-27].

Though residents were satisfied with their training program overall, there are various areas for improvement. Our most remarkable finding was that even though residents thought that the curriculum was too short, they nonetheless expected to be able to function effectively in their future roles as EPs. It is not yet known whether they will maintain this view once they have started as EPs. Future research should examine whether international comparisons of either emergency medicine alone or residents’ perspectives on it are possible. We intend to adjust our questionnaire and examine how emergency physicians assess their own performance. It is essential for subsequent research to explore whether Dutch EPs meet international standards.

## Conclusion

EM in The Netherlands is a recent medical specialty: inevitably, it is still developing. As the 3-year training program was recognized only in November 2008, it too is still under continuous development. We conducted a national survey to evaluate it for the first time and identified areas that its residents believed should be improved.

The EM training program should be extended to 5 years. The compulsory nature of the general practice rotation should be reconsidered. Training programs should be provided in hospitals where EPs are continuously available for supervision and bedside teaching. The educational research program undertaken as part of the training program should be more structured and better embedded. Greater attention should be paid to embedding skills such as PSA and FAST in the national training program.

At the same time, residents expected that they would be able to function effectively as EPs once they had finished the current training program. We conclude that, from the residents’ perspective, the Dutch EM curriculum has solid foundations, but that there are also areas for improvement.

## Abbreviations

CanMED: Canadian Medical Education Directives for Specialists; CAT: Critical appraisal of a topic; ED: Emergency department; EM: Emergency medicine; EP: Emergency physician; EuSEM: European Society for Emergency Medicine; FAST: Focused assessment with sonography in trauma; KNMG: Royal Dutch Medical Association; MSRC: Medical Specialist Registration Committee; NVSHA: Netherlands Society of Emergency Physicians; OSAT: Objective structured assessment of technical skill; PSA: Procedural sedation and analgesia.

## Competing interests

There are no financial or other conflicts of interest.

## Authors’ contributions

All authors contributed to the research and work presented in this article. As first author, SK drafted and distributed the questionnaire, collected and processed the completed questionnaires, drafted and distributed the reminder letter, performed the statistical analyses and co-wrote the manuscript. The initiative for the study lay with MG, the second author, who also drafted the questionnaire and reminder letter, and co-wrote the manuscript. RV, the third author and primary supervisor, drafted the questionnaire and supervised and contributed to writing the manuscript. All authors read and approved the final manuscript.

## References

[B1] Gaakeer MI van den Brand CL Patka P Emergency medicine in the Netherlands: a short history provides a solid basis for future challenges Eur J Emerg Med 2012 6 131 135 22293304 10.1097/MEJ.0b013e3283509d94

[B2] Holmes JL Emergency medicine in the Netherlands Emerg Med Australas 2010 6 75 81 20152006 10.1111/j.1742-6723.2009.01259.x

[B3] Royal Dutch Medical Association Besluit Spoedeisende geneeskunde 2013 7 1 http://knmg.artsennet.nl/Opleiding-en-Registratie/Algemene-informatie/Nieuws/O-R-Nieuwsartikel/Vaststelling-van-het-Besluit-Spoedeisende-geneeskunde.htm

[B4] Gijsen R Kommer G Bos N Stel Van H Hoe groot is het gebruik van de afdeling Spoedeisende hulp? 2012 13 12 http://www.nationaalkompas.nl/zorg/sectoroverstijgend/acute-zorg/spoedeisende-hulp/hoe-groot-is-het-gebruik-van-de-afdeling-spoedeisende-hulp/

[B5] Alkemade AJ Van Driel A Geijsel FEC Ter Maarten JC Schouten I Curriculum opleiding tot Spoedeisende Hulp Arts 2008 13 18 http://knmg.artsennet.nl/web/file?uuid=3320a5b3-0188-4d08-8107-a44879e08fb6&owner=a8a9ce0e-f42b-47a5-960e-be08025b7b04&contentid=54977&elementid=1787161 20356436

[B6] Royal College of Physicians and Surgeons of Canada The CanMEDS Framework 2005 http://www.royalcollege.ca/portal/page/portal/rc/canmeds

[B7] EuSEM Taskforce on Curriculum European Curriculum For Emergency Medicine 2009 25 4 http://www.eusem.org/cms/assets/1/pdf/european_curriculum_for_em-aug09-djw.pdf

[B8] Aksay E Sahin H Kiyan S Ersel M Current status of emergency residency training programs in Turkey: after 14 years of experience Eur J Emerg Med 2009 6 4 10 18931617 10.1097/MEJ.0b013e32830a7553

[B9] Ahmad S De Oliveira GSJ McCarthy RJ Status of anesthesiology resident research education in the United States: structured education programs increase resident research productivity Anesth Analg 2013 6 205 210 23223116 10.1213/ANE.0b013e31826f087d

[B10] Aldeen AZ Gisondi MA Bedside teaching in the emergency department Acad Emerg Med 2006 6 860 866 16766739 10.1197/j.aem.2006.03.557

[B11] De Witt C Jr B Daugherty SR Ryan PM How Residents View Their Clinical Supervision: A Reanalysis of Classic National Survey Data Journal of Graduate Medical Education: March 2010 2010 6 1 37 45 10.4300/JGME-D-09-00081.1 PMC2931227 21975882

[B12] Celenza A Evolution of emergency medicine teaching for medical students Emerg Med Australas 2006 6 219 220 16712530 10.1111/j.1742-6723.2006.00862.x

[B13] Craig S Direct observation of clinical practice in emergency medicine education Acad Emerg Med 2011 6 60 67 21175927 10.1111/j.1553-2712.2010.00964.x

[B14] Farnan JM Petty LA Georgitis E Martin S Chiu E Prochaska M A systematic review: the effect of clinical supervision on patient and residency education outcomes Acad Med 2012 6 428 442 22361801 10.1097/ACM.0b013e31824822cc

[B15] Kilroy DA Clinical supervision in the emergency department: a critical incident study Emerg Med J 2006 6 105 108 16439737 10.1136/emj.2004.022913 PMC2564027

[B16] Vardy JM Dignon N Mukherjee N Sami DM Balachandran G Taylor S Audit of the safety and effectiveness of ketamine for procedural sedation in the emergency department Emerg Med J 2008 6 579 582 18723707 10.1136/emj.2007.056200

[B17] Metzner J Domino KB Risks of anesthesia or sedation outside the operating room: the role of the anesthesia care provider Curr Opin Anaesthesiol 2010 6 523 531 20531171 10.1097/ACO.0b013e32833b7d7c

[B18] Ramaiah R Bhananker S Pediatric procedural sedation and analgesia outside the operating room: anticipating, avoiding and managing complications Expert Rev Neurother 2011 6 755 763 21539491 10.1586/ern.11.52

[B19] O’Connor RE Sama A Burton JH Callaham ML House HR Jaquis WP Procedural sedation and analgesia in the emergency department: recommendations for physician credentialing, privileging, and practice Ann Emerg Med 2011 6 365 370 21802778 10.1016/j.annemergmed.2011.06.020

[B20] Molina JA Lobo CA Goh HK Seow E Heng BH Review of studies and guidelines on fasting and procedural sedation at the emergency department Int J Evid Based Healthc 2010 6 75 78 20923510 10.1111/j.1744-1609.2010.00163.x

[B21] Kuypers MI Mencl F Verhagen MF Kok MF Dijksman LM Simons MP Safety and efficacy of procedural sedation with propofol in a country with a young emergency medicine training program Eur J Emerg Med 2011 6 162 167 21164345 10.1097/MEJ.0b013e32834230fb

[B22] Arafat R Golea A Daramus I Badea R Medical education for emergency physician focused on basic competence (focused assessment with sonography in trauma): evaluation of the Romanian national program: “regional emergency medical services systems” Med Ultrason 2011 6 283 291 22132400

[B23] Brenchley J Walker A Sloan JP Hassan TB Venables H Evaluation of focussed assessment with sonography in trauma (FAST) by UK emergency physicians Emerg Med J 2006 6 446 448 16714505 10.1136/emj.2005.026864 PMC2564340

[B24] Lanoix R Leak LV Gaeta T Gernsheimer JR A preliminary evaluation of emergency ultrasound in the setting of an emergency medicine training program Am J Emerg Med 2000 6 41 45 10674530 10.1016/s0735-6757(00)90046-9

[B25] Ma OJ Mateer JR Ogata M Kefer MP Wittmann D Aprahamian C Prospective analysis of a rapid trauma ultrasound examination performed by emergency physicians J Trauma 1995 6 879 885 7602628 10.1097/00005373-199506000-00009

[B26] Ma OJ Gaddis G Norvell JG Subramanian S How fast is the focused assessment with sonography for trauma examination learning curve? Emerg Med Australas 2008 6 32 37 18062785 10.1111/j.1742-6723.2007.01039.x

[B27] Mahler SA Swoboda TK Wang H Arnold TC Dedicated emergency department ultrasound rotation improves residents’ ultrasound knowledge and interpretation skills J Emerg Med 2012 6 129 133 21550756 10.1016/j.jemermed.2011.03.028

